# Pensions for Physicians

**Published:** 1916-09

**Authors:** 


**A Monthly Review of Medicine and Surgery**

EDITOR AND PUBLISHER, DR. A. L. BENEDICT, 228 Summer St., corner of Elmwood Ave., Buffalo, (Address for all communications. Please make personal and telephone calls before 1 P. M.)

Third, in age, on the western continent. Independent, but supports professional organizations. News limited mainly to Western N. Y. and Penn.

Subscription $2.00 a year; $3.00 for two years in advance. Changes and errors in address or failure or duplication of delivery, should be reported immediately.

Yearly Volume 72 SEPTEMBER, 1916 Number 2

### Pensions for Physicians.

Too few replies have been received on this subject to present them at present and we earnestly ask for expressions of opinion. It has been intimated, however, that a modest beginning of a fund is in sight. Meantime, a phase of the matter has been presented which was not contemplated but which is of considerable importance. In medicine as in other sciences, certain men make discoveries or perfect inventions of great value to the race but either not susceptible of being distributed so as to afford a living for the discoverer or requiring mental attributes and methods not compatible with earning a living. To such men, the world owes a debt not merely of gratitude but of practical maintenance somewhat conforming to the value of the service to humanity. Yet, in many instances, the discovery or invention is exploited so that its originator gets nothing or very little from his labors while not even the public benefits from it except remotely. This phase of recognition of services involves something more than the relief, in old age or incapacity of the routine worker and it requires, for the adequate discharge of the obligation, different means from those contemplated. It can not be satisfactorily carried out by a few world prizes, nor by the present patent laws, even disregarding the objection to patents of medical devices, nor by private annuities. Without having been able to give the problem the consideration which it deserves, it occurs to us that the nation should legalize the principle of rewarding great discoveries in any line, in cases in which the discovery is so broad and so intangible that a patent is out of the question; or, in lieu of a patent, should buy the invention whenever the government can control or employ it directly and conveniently or should pay an honorarium in other instances, if it be thrown open to general manufacture and open competition. Any such legislation would require some sort of board of investigation and appraisal, of permanent nature.

